# Molecular Tools for Typing *Mycoplasma pneumoniae* and *Mycoplasma genitalium*

**DOI:** 10.3389/fmicb.2022.904494

**Published:** 2022-06-02

**Authors:** Roger Dumke

**Affiliations:** TU Dresden, Institute of Medical Microbiology and Virology, Dresden, Germany

**Keywords:** *Mycoplasma pneumoniae*, *Mycoplasma genitalium*, molecular typing, epidemiology, SNP, MLST, tandem repeats

## Abstract

*Mycoplasma pneumoniae* and *Mycoplasma genitalium* are cell wall-less bacteria with strongly reduced genome content and close phylogenetic relatedness. In humans, the only known natural host, the microorganisms colonize the respiratory or genitourinary mucosa and may cause a broad range of clinical presentations. Besides fundamental differences in their tissue specificity, transmission route, and ability to cause prevalence peaks, both species share similarities such as the occurrence of asymptomatic carriers, preferred populations for infection, and problems with high rates of antimicrobial resistance. To further understand the epidemiology of these practically challenging bacteria, typing of strains is necessary. Since the cultivation of both pathogens is difficult and not performed outside of specialized laboratories, molecular typing methods with adequate discriminatory power, stability, and reproducibility have been developed. These include the characterization of genes containing repetitive sequences, of variable genome regions without the presence of repetitive sequences, determination of single and multi-locus variable-number tandem repeats, and detection of single nucleotide polymorphisms in different genes, respectively. The current repertoire of procedures allows reliable differentiation of strains circulating in different populations and in different time periods as well as comparison of strains occurring subsequently in individual patients. In this review, the methods for typing *M. pneumoniae* and *M. genitalium*, including the results of their application in different studies, are summarized and current knowledge regarding the association of typing data with the clinical characteristics of infections is presented.

## Introduction

During the evolutionary interplay with their hosts, the genomes of species of the class Mollicutes (“mycoplasma”) have been greatly reduced. Besides limited metabolic capabilities, the lack of a classical bacterial cell wall is the most striking result of this interaction. In humans, different species can be found as commensals whereas the most clinically relevant *Mycoplasma pneumoniae* and *Mycoplasma genitalium* are host-specific pathogens that infect the respiratory and genitourinary mucosa. *Mycoplasma pneumoniae* is transmitted *via* contaminated aerosols and is a frequent cause of community-acquired respiratory tract infections including severe cases of interstitial pneumonia (Khoury et al., 2016). Infections can occur in all age groups but school-aged children are the preferred population. Besides small-scale outbreaks in settings with close person-to-person contacts, such as military camps or schools (Waites et al., 2017), epidemic peaks are registered every 3–7 years (Brown et al., 2016; Kenri et al., 2020). During these periods, which sometimes can be registered worldwide, *M. pneumoniae* may cause up to 50% of all community-acquired respiratory infections (Ho et al., 2015; Kogoj et al., 2015). In addition, a broad spectrum of extrapulmonary manifestations is described, mainly affecting the central nervous system and the skin (Narita, 2016). In contrast, *M. genitalium* is a sexually transmitted pathogen that causes non-gonococcal urethritis in men and is associated with urethritis, cervicitis, endometritis, and pelvic inflammatory disease in women (Jensen et al., 2022). The prevalence of infection ranges between 1.3 and 3.9% in the general population (Baumann et al., 2018) but can be significantly higher in risk groups such as men who have sex with men (MSM) and HIV-positive patients (Jansen et al., 2020; Latimer et al., 2020).

Treatment of infections with both species is challenging as mycoplasmas are intrinsically resistant to betalactams. Generally, tetracyclines and quinolones are effective but have side effects for relevant patient groups (treatment of *M. pneumoniae* in pediatrics) or are of limited clinical efficacy (*M. genitalium* and doxycycline). Hence, macrolides are the antibiotics recommended primarily for treatment of adults and children with severe clinical disease and in attempts at eradication in symptomatic or asymptomatic patients. Unfortunately, high rates of acquired resistance to macrolides (*M. pneumoniae*) and to macrolides and quinolones (*M. genitalium*) are described (Fernandez-Huerta et al., 2020a; Machalek et al., 2020; Pereyre and Tardy, 2021). Despite strong epidemiological differences, such as the location of colonized tissues and consequently the transmission route of infections, there are similarities in epidemiological characteristics. Besides the high rates of resistance, these include the occurrence of asymptomatic carriers probably able to transmit the pathogens (Spuesens et al., 2013; Gnanadurai and Fifer, 2020; de Groot et al., 2021).

As is typical for mycoplasmas, genomes of species are small but closely related phylogenetically, with around 580 kbp (*M. genitalium*) and 816 kbp (*M. pneumoniae*). Many orthologous proteins (~480) can be found in both pathogens and identity between them has been calculated as around 67% (Himmelreich et al., 1997). In the last few years, whole genome sequencing of isolates of different geographic origin and from varying time periods resulted in strong similarities between compared strains (>99%) and classification into two main lineages. Within these main genotypes, the genomes of both species were identical in >99.5%, which is remarkably high (Lluch-Senar et al., 2015; Xiao et al., 2015; Diaz et al., 2017; Fookes et al., 2017; Lee et al., 2019). Moreover, construction of phylogenetic trees of *M. pneumoniae* genomes resulted in different clades within the main types showing differences in the frequency of *in vivo* occurrence and in the number of recombination events (Kenri et al., 2020; Hsieh et al., 2022). Despite the overall conserved genomes, regions with higher heterogeneity were shown to be associated in most cases with the occurrence of repetitive elements distributed in similar but non-identical copies in the genomes. Four repetitive elements (RepMp1, 2/3, 4, and 5) were found in *M. pneumoniae*, whereas repeated sequences from *M. genitalium* cluster in nine discrete regions are known as MgPar. Genome parts with copies of repetitive elements can be exchanged by homologous recombination (Musatovova et al., 2008; Spuesens et al., 2009; Hakim et al., 2021). This also applies to genes coding for surface-localized and antigenic proteins with special importance for the infection process such as the adhesins P1 and P40/P90 from *M. pneumoniae*, and MgpB and MgpC from *M. genitalium* (also known as P140 and P110). Resulting modifications of these proteins has been postulated as immune escape mechanism of the bacteria (Rocha and Blanchard, 2002; Hakim et al., 2021).

To further understand the epidemiology of infections by *M. pneumoniae* and *M. genitalium*, typing of strains is important. Unfortunately, cultivation of both species is difficult and only realized in few specialized centers. Therefore, molecular detection is common in routine laboratories and positive DNA from clinical material is the most frequent specimen available for typing in practice.

## Development of Typing Methods

The first molecular method for typing *M. pneumoniae* isolates was PCR-mediated DNA fingerprinting, which confirmed that there were two main strain types (Su et al., 1990; Ursi et al., 1994). Supported by data from early whole genome sequencing of *M. pneumoniae* (type 1 strain M129; Himmelreich et al., 1996), more targeted methods were introduced in the following years. These included the restriction fragment length polymorphism procedure, which uses primers that amplify both regions of the *p1* gene (MPN141) containing copies of the repetitive elements RepMP2/3 and RepMP4 (Sasaki et al., 1996). This approach distinguished the two main *p1* types and a limited number of additional genotypes (Kenri et al., 2020). To detect all sequence variations, the more laborious amplification of RepMp copies in the *p1* gene followed by Sanger sequencing has been used (Dumke et al., 2006, 2015).

In 2009, multi-locus variable-number tandem-repeat analysis (MLVA) was introduced using five loci [HsdS (MPN089), intergenic, and hypothetical proteins (MPN501, MPN524, and MPN613)] to determine the number of repeats (Degrange et al., 2009). The results of typing of isolates showed an excellent discriminatory index (DI) of 0.92. The method was adapted for investigation of *M. pneumoniae*-positive DNA (Dumke and Jacobs, 2011). Unfortunately, the locus with the highest DI (Mpn1, coding for a subunit of type I restriction-modification enzyme, MPN089) was found to be instable (Benitez et al., 2012; Sun et al., 2013) and must be removed from the list of repeats. For the remaining four repeats, interpretation guidelines for use of MLVA were established to allow reliable interlaboratory comparison of results (Chalker et al., 2015). In some strains, differences in the length of distinct tandem repeat loci must be considered (Xue et al., 2018; Kenri et al., 2020). Further tandem repeats were tested and might be an alternative to Mpn1 to increase the discrimination of the method (Zhang et al., 2017). In addition, AGT repeats can be found in the region between the repetitive elements in the *p1* gene. Using this single locus of tandem repeats, strains with identical MLV type were differentiated and the number of *p1* repeats is associated with the main *p1* types (Zhao et al., 2011; Tian et al., 2013; Xiao et al., 2020). Unfortunately, there is evidence that this marker is unstable and this needs further analysis (Spuesens et al., 2016; Dumke, unpubl.). Recently, a new target of VNTR analysis was reported using the tandem repeats in subunit S of the type I restriction-modification system (MPN085) of *M. pneumoniae* for differentiation of strains (Lee et al., 2022).

Based on comprehensive comparison of the increasing number of whole genome data, two methods that use determination of single nucleotide polymorphisms (SNPs) for typing were developed. SNPs in eight genes (MPN003, MPN185, MPN246, MPN307, MPN528, MPN576, MPN600, and MPN628) coding for house-keeping proteins (Brown et al., 2015; here called MLS typing) or for house-keeping proteins (MPN004, MPN050, MPN168, MPN246, and MPN516), hypothetical lipoproteins (MPN442, MPN582), and the P1 adhesin [(MPN141); Touati et al., 2015; SNP typing] were selected. Both methods can be used not only for characterization of isolates but also for investigation of strains in DNA-positive clinical samples (Dumke and Rodriguez, 2021) and result in a numerical code or a SNP profile, which can be easily exchanged between laboratories. For a high discriminatory power of differentiation if MLS and SNP typing is performed in parallel, use of same loci [gmk (MPN246)] is disadvantageous. In addition, SNP measured in the *p1* gene (SNP typing) is located in the repetitive element RepMp2/3 and homologous recombination of this locus cannot be excluded. For the method of Brown et al., a database was established that comprises listing, consecutive numbering, and comparison of detected MLS types.^1^

In addition to the aforementioned methods, various other typing methods were developed but are not widely used. Real-time PCR with high-resolution melting point analysis is able to differentiate *p1* type 1 and 2 strains (Schwartz et al., 2009). Moreover, pyrosequencing of two targets (MPN141 and MPN528a) resulted in correct classification of both main *p1* types (Spuesens et al., 2010). The MPN142 gene (historically named ORF6) contains a copy of the repetitive element RepMp5 and amplification/sequencing can be used to distinguish the main types 1 and 2 as well as some, but not all, other *p1* types (Ruland et al., 1994; Kenri et al., 2020). Investigation of the protein composition of bacteria by MALDI-ToF is not only suitable for reliable characterization of mycoplasmas on the species level but also for differentiation of the two main types of *M. pneumoniae* (Pereyre et al., 2013; Xiao et al., 2014). Of note, the procedure is described for investigation of isolates. Finally, nanorod array surface-enhanced Raman spectroscopy was used for detection of *M. pneumoniae* and typing of isolates and strains in clinical throat swabs (Hennigan et al., 2010; Henderson et al., 2015). Special equipment and experienced staff are required for this typing method which is, therefore, reserved for specialized laboratories.

In comparison with *M. pneumoniae*, the number of procedures for typing *M. genitalium* strains is relatively small. The first method was investigation of a variable part of the MG_191 gene, which codes for the adhesin MgpB (Hjorth et al., 2006). This gene contains repetitive elements but the typing region near the 5′ end (nt 180–460 in type strain G37) was found to be stable during *in vivo* and *in vitro* passage of isolates and is not influenced by homologous recombination. The results of the study by Hjorth et al. (2006) confirmed not only the sexual transmission of *M. genitalium* between couples but also the usefulness of culture-independent *mgpB* typing to investigate the circulation of genotypes in different populations and to characterize strains in cases of treatment failure. Furthermore, analysis of different short tandem repeats in the gene MG_309, which codes for a surface-localized lipoprotein, is suitable for typing (Ma and Martin, 2004; Ma et al., 2008; McGowin et al., 2009). Combining the number of these repeats (AGT/AAT) with *mgpB* typing increases the discriminatory power. Further targets of strain discrimination as well as MLVA testing were found to be not stable, not discriminatory enough, too discriminatory (Cazanave et al., 2012) or were used in a very limited number of studies (Ma and Martin, 2004; Pineiro et al., 2019) to date.

## Evaluation of Common Typing Methods

A sufficiently large number of data for comparison of the results of different methods are available for *p1*, MLV, MLS, and SNP typing in *M. pneumoniae* and for *mgpB* and MG_309 typing in *M. genitalium* (Table 1). Using *p1* typing, calculated DI’s (Hunter and Gaston, 1988) from selected studies ranged between 0.42 and 0.68. To date, 15 *p1* types have been described (Kenri et al., 2020; Xiao et al., 2020). The main types 1 and 2 contain specific repetitive sequences in their genomes, suggesting early differentiation of *M. pneumoniae* strains into these two lineages (Spuesens et al., 2009; Diaz and Winchell, 2016). In contrast, the 13 known variants can be assigned to the main genotypes but differ in one or both repetitive copies in the *p1* gene. Criteria for recognizing a strain as variant should be set in the future (e.g., length of sequence which must be different from known *p1* types to define a new type, deposition of full-length sequences of both RepMP2/3, and four elements of *p1* gene of the strain in databases). Interestingly, by contrast with *p1* type 1, more variant strains have been characterized among type 2. This might be attributed to type-specific differences in the functionality of proteins putatively involved in DNA recombination and repair in *M. pneumoniae* and *M. genitalium* (Sluijter et al., 2010; Hakim et al., 2021).

**Table 1:** Characteristics of frequently used molecular approaches for typing *Mycoplasma pneumoniae* and *Mycoplasma genitalium* (results of studies with >25 patients are included; if not presented in the study, HGDI’s are calculated according to the data).

Species	Typing method	Number of known types	HGDI	References
*M. pneumoniae*	*p1*	15	0.42–0.68	Brown et al., 2015; Dumke et al., 2015; Touati et al., 2015; Kenri et al., 2020; Xiao et al., 2020; Dumke and Rodriguez, 2021; Meyer Sauteur et al., 2021
	MLVA	24	0.58–0.68	Benitez et al., 2012; Brown et al., 2015; Dumke et al., 2015; Touati et al., 2015; Kogoj et al., 2018; Zhao et al., 2019; Kenri et al., 2020; Xiao et al., 2020; Dumke and Rodriguez, 2021; Meyer Sauteur et al., 2021
	MLST	46	0.66–0.78	http://pubmlst.org/mpneumoniae; Brown et al., 2015; Kenri et al., 2020; Dumke and Rodriguez, 2021; Meyer Sauteur et al., 2021
	SNP	15	0.80–0.84	Touati et al., 2015; Kenri et al., 2020; Dumke and Rodriguez, 2021
	*p1* + MLVA; *p1* + MLST; *p1* + SNP; MLVA + SNP; and MLST + SNP	n.d.	0.60–0.88	Brown et al., 2015; Dumke et al., 2015; Touati et al., 2015; Kenri et al., 2020; Xiao et al., 2020; Dumke and Rodriguez, 2021; Meyer Sauteur et al., 2021
	*p1* + MLVA + MLST; *p1* + MLVA + SNP	n.d.	0.75–0.91	Brown et al., 2015; Touati et al., 2015; Kenri et al., 2020; Dumke and Rodriguez, 2021; Meyer Sauteur et al., 2021
	*p1* + MLVA + MLST + SNP	n.d.	0.88–0.90	Kenri et al., 2020; Dumke and Rodriguez, 2021
*M. genitalium*	*mgpB*	246	0.82–0.99	Hjorth et al., 2006; Ma et al., 2008; Cazanave et al., 2012; Olsen et al., 2012; Dumke et al., 2020; Fernandez-Huerta et al., 2020b; Plummer et al., 2020; Sweeney et al., 2020; Chua et al., 2021; Dumke and Spornraft-Ragaller, 2021; Guiraud et al., 2021
	VNTR MG_309	12	0.84–0.95	Ma et al., 2008; Cazanave et al., 2012; Dumke and Spornraft-Ragaller, 2021; Guiraud et al., 2021; Laumen et al., 2021
	*mgpB* + VNTR MG_309	n.d.	0.95–0.99	Ma et al., 2008; Cazanave et al., 2012; Dumke and Spornraft-Ragaller, 2021; Guiraud et al., 2021

p1, sequence differences in p1 gene (MPN141); MLVA, multilocus variable number of tandem-repeat analysis; MLST, multilocus sequence typing; SNP, determination of single polynucleotide polymorphisms; mgpB, sequence differences in *mgpB* gene (MG_191); VNTR MG_309, variable number of tandem repeat in gene MG_309; HGDI, discriminatory index (Hunter and Gaston, 1988); and n.d., not determined.

**Figure 1:**
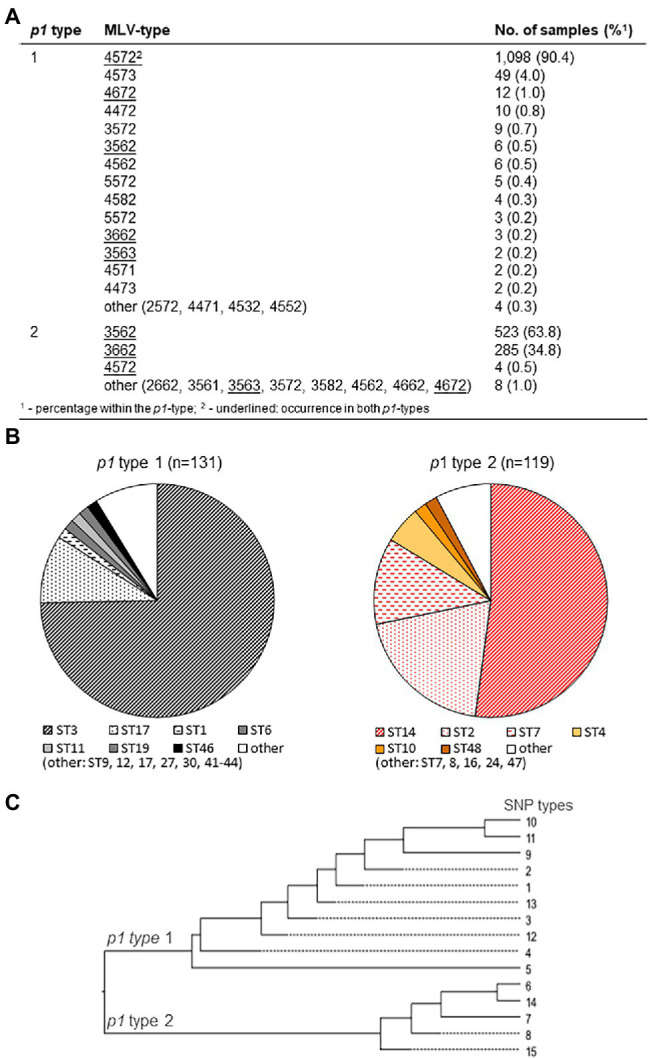
Association of main *p1* types 1 and 2 (including variants) with multi-locus variable-number (MLV; **A**; using data of Benitez et al., 2012; Brown et al., 2015; Dumke et al., 2015; Touati et al., 2015; Kogoj et al., 2018; Zhao et al., 2019; Kenri et al., 2020; Xiao et al., 2020; Dumke and Rodriguez, 2021; Meyer Sauteur et al., 2021), MLS (**B**; data of Brown et al., 2015; Kenri et al., 2020; Meyer Sauteur et al., 2021; and Dumke and Rodriguez, 2021), and single nucleotide polymorphism (SNP) types (**C**; data of Touati et al., 2015; Kenri et al., 2020; Dumke and Rodriguez, 2021), respectively.

As an example for the occurrence of MLV types in various populations, the results of selected studies (characterization of >50 strains) in different countries are summarized in Table 1 and Figure 1A. With at least 24 known types, MLVA demonstrated a greater number of genotypes in comparison with *p1* typing. However, calculated DI’s in these reports are not substantially higher due to the strong dominance (94%) of the three MLV types 4,572, 3,562, and 3,662, respectively. After review of further studies, additional MLV types were found in a small number of strains, underlining the need to summarize the typing results in an appropriate manner (preferably in a database). In contrast, 46 MLS types have been registered in the corresponding database and recent studies using MLST resulted in DI’s between 0.66 and 0.78, respectively. Higher DI’s up to 0.84 were calculated for SNP typing despite the fact that the overall number of SNP types is relatively low. However, it should be mentioned that this method was used to a lesser extent in comparison with the other typing methods up to now. Hence, data on the discriminatory power of this method might be preliminary.

According to the calculated DI’s, adequate discrimination of *M. pneumoniae* strains (DI ≥ 0.9) can only be achieved if different typing methods are used. For selected examples (reports in which typing results for all strains are listed), the discrimination power of method combinations was calculated in Table 1. In practice, such an approach is laborious and the volume of DNA obtained after use of automated systems for DNA preparation from clinical specimens could be too low to perform three or four typing procedures in parallel.

As the genomes of strains belonging to one of the two main lineages of *M. pneumoniae* are strongly related, an association of *p1* types 1 and 2 with genotypes identified by other typing methods is probable. As summarized in Figure 1A, many MLV types (79%) can be assigned exclusively to one of the main genotypes. In contrast, five MLV types occurred in both main lineages but in most cases (like 4,572, 3,562, and 3,662) with a clear numerical preference for a *p1* type. As errors in counting the tandem repeats cannot be excluded, consideration of guide line for VNTR typing of *M. pneumoniae* (Chalker et al., 2015) is strongly recommended. Although the number of strains with a MLV type not belonging to the preferred *p1* type is low (0.9%), MLV typing cannot be used to replace *p1* typing. In contrast, the known MLS and SNP types are in complete agreement with *p1* types (Figures 1B,C).

Regarding *M. genitalium*, *mgpB* typing has resulted in a large number of types, confirming the variability of the region of *mgpB* gene used. Nearly 250 *mgpB* types have now been described (Table 1). To answer epidemiological questions regarding the distribution of strains in different human populations, the stability and strong heterogeneity of the typing region allow reliable characterization of isolates. The corresponding DI’s for *mgpB* sequencing in different studies varied between 0.82 and 0.99 whereas MG_309 typing resulted in discrimination between 0.84 and 0.95. Lower DI’s seem to be associated with the investigation of more local than national populations (Dumke et al., 2020; Dumke and Spornraft-Ragaller, 2021), indicating a lower number of circulating genotypes. DI’s of ≥0.95 were obtained after the *mgpB* and MG_309 methods were combined. With these values, further optimization of typing for *M. genitalium* does not seem necessary. For standardized comparison of *mgpB* sequences, the defined part of the gene (position 221,749–222,029 in the genome of strain G37, GenBank no. NC_000908.2) should be fully sequenced. For example, when analyzing several deposited sequences, differentiation between type 2 and 74 is not possible due to their short lengths. Additionally, determination of the number of tandem repeats at locus MG_309 can be complicated in practice by the presence of mixed sequences in some samples. Careful inspection of the results after sequencing in both directions can help to solve this problem (Ma et al., 2008).

## Association of Typing Results With Epidemiological and Clinical Parameters of Infections

Besides the use of typing to elucidate the epidemiological correlations of infections due to *M. pneumoniae* and *M. genitalium*, relating typing results to clinically relevant aspects is of practical importance for clinicians. These include associations between genotypes and severity of clinical disease, distinct symptoms of infection, site of infection, or antibiotic resistance. Regarding *M. pneumoniae*, the association between typing results and clinical aspects has been investigated in only a few studies. In comparison with other MLVA types, a statistically significantly higher pneumonia severity index, longer duration of cough, and older age of patients were demonstrated after infection with the mainly *p1* 1-specific type 4/5/7/2 (Qu et al., 2013). In a further study, a higher rate of severe pneumonia was demonstrated if children were infected with *p1* type 1 vs. type 2 (Fan et al., 2017). In contrast, Yan et al. (2019) reported a higher rate of pediatric patients with pleural effusion as a severe complication of *M. pneumoniae* pneumonia after infection with MLVA type 3/5/6/2 (mainly *p1* type 2) vs. infection with type 4/5/7/2. In a study among Slovenian children, infection with *p1* type 2 strains resulted in an elevated C-reactive protein level and a higher rate of hospital admissions in comparison with *p1* type 1 infections (Rodman Berlot et al., 2021). Regarding extrapulmonary manifestations of infections, only very few reports have dealt with the possible influence of the genotype on this complex of diseases. In the case of *M. pneumoniae*-induced mucocutaneous disease (Steven-Johnson syndrome), a rare but severe complication of infection, genotype does not seem to be a determinant of clinical symptoms (Olson et al., 2015; Watkins et al., 2017; Meyer Sauteur et al., 2021). In conclusion, the limited and partly heterogeneous results underline the need for further clinical studies with well-characterized patient populations of different age to confirm or exclude correlations of genotypes with clinical manifestations and outcomes of *M. pneumoniae* infections. This should include investigation of the pathogen in the lower and upper respiratory tract of patients, which might influence the ratio of *p1* genotypes (Xiao et al., 2020). Furthermore, typing studies among symptomatic patients and asymptomatic carriers would be helpful to clarify if genotypes play a role in the differences in the clinical manifestation of *M. pneumoniae* infections between the groups (Spuesens et al., 2013; de Groot et al., 2021). In addition, further genetic, proteomic, and phenotypic investigations will help to understand differences between genotypes, which might explain clinical aspects of infections. To date, the main *p1* types vary with regard to the development of biofilms (Simmons et al., 2013) and expression of the CARDS toxin (Lluch-Senar et al., 2015), which is a virulence factor with special relevance in pathogenesis (Su et al., 2021).

**Figure 2:**
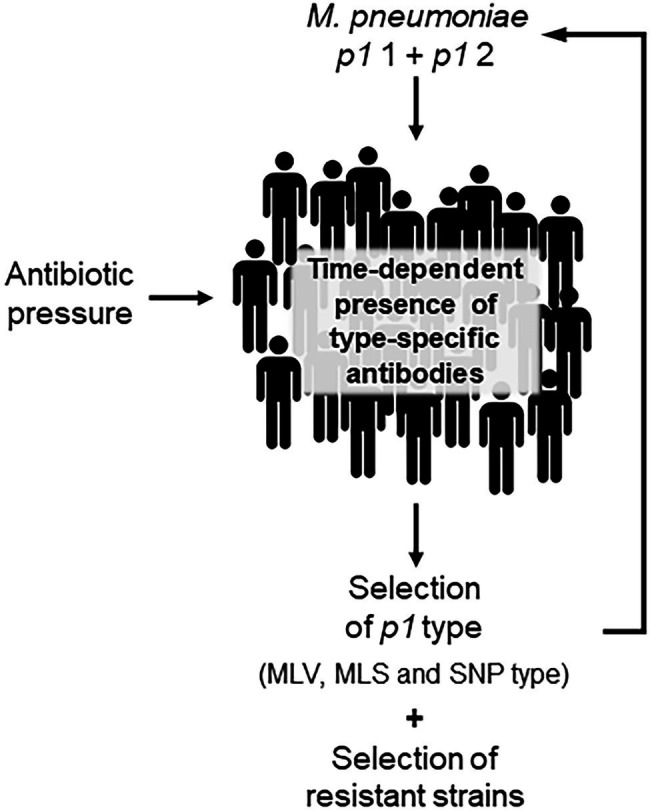
Schematic illustration of time-dependent shift of *Mycoplasma pneumoniae* genotypes.

Circulation of different *p1* types in the human population has been suggested to explain the typical epidemiology of infections as the immunodominant P1 protein is crucial for adhesion of bacteria to the cells of the respiratory epithelium as the first and essential stage in clinical manifestation. Regions of this adhesin, which contains repetitive sequences, were characterized as surface-located and it can be assumed that type-specific antibodies will be produced during host colonization (Dumke et al., 2008; Schurwanz et al., 2009; Nakane et al., 2011; Vizarraga et al., 2020). If these antibodies play a role in the adherence process, their quantitative occurrence in a host population could influence the distribution of type 1 or 2 strains (Figure 2). Based on the time-dependent level of herd immunity, the reported type shifts of *p1* types in combination with an increase in the prevalence of infection might be explained by the presence of and change in type-specific antibodies. Studies have confirmed the polyclonality of strains in nation-wide investigations as a precondition for changes of genotypes and a varying dominance of type 1 or 2 in different regions (Kogoj et al., 2018; Lee et al., 2018) as well as a time period of 5–10 years for type change in Japan (Kenri et al., 2020). In some cases, the temporary dominance of a *p1* type reaches more than 90% (Sun et al., 2017; Kenri et al., 2020). In contrast, if levels of type-specific antibodies do not differ greatly, the occurrence of both *p1* types without dominance of one lineage is demonstrated (Jacobs et al., 2015; Xiao et al., 2020; Guo et al., 2022). Further studies are needed to confirm the association between type and type-specific antibodies experimentally (Dumke et al., 2010). However, results of mathematical models support the hypothesis of co-circulation of both *p1* types and the importance of herd immunity for the ratio of genotypes (Omori et al., 2015; Zhang et al., 2019). This pattern seems primarily independent of the rate of resistance in the corresponding population. Despite the regional emergence of types with high rates of macrolide resistance (Ho et al., 2015; Lee et al., 2018; Hung et al., 2021), a clear association of resistance with distinct genotypes of *M. pneumoniae* was not found. Recently, an association between the number of tandem repeats in subunit S of the type I restriction-modification system (MPN085) and macrolide resistance in MLST-3 strains was reported (Lee et al., 2022). This interesting aspect should be investigated in future studies including further types. It can be assumed that the regional/national use of macrolides and the treatment regime in particular patients will be crucial for the development of resistant strains. Up to now, there is no *in vivo* or *in vitro* evidence for a greater rate of resistance among the described genotypes. Thus, antibiotic pressure in a population will lead to an increasing rate of resistance in the dominating type(s), which is primarily determined by the circulation of *p1* types and, due to the association with the main *p1* types, secondarily by the regional occurrence of MLV, MLS, or SNP types (Figure 1). Consequently, unsubstantiated use of macrolides in combination with the lack of resistance-guided therapy regimes in many settings worldwide will result in the selection of resistant strains. This hypothesis is supported by the results of studies reporting a decrease in the resistance rate after genotype change (Nakamura et al., 2021), followed by a subsequent increase among strains of the previously nondominant genotype (Wang et al., 2021). In small-scale outbreaks, time-dependent emergence of resistance among strains of the same genotype can be found (Hubert et al., 2021), emphasizing the need for mutation analysis in cases of treatment failure.

**Figure 3:**
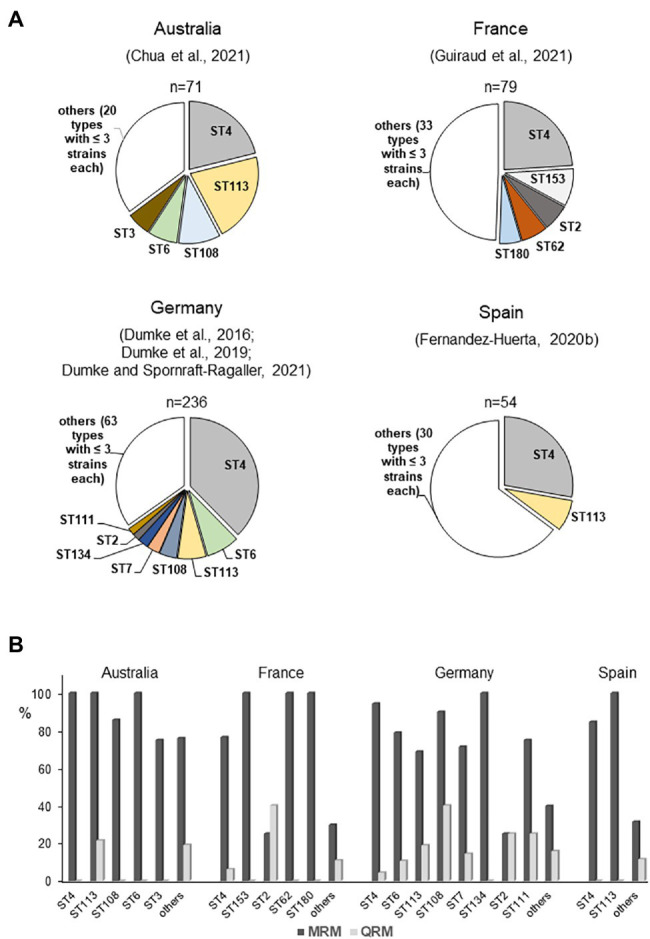
Regional distribution of *mgpB* types **(A)** and association between *mgpB* type and resistance **(B)** among strains in selected studies included mainly men who have sex with men (MSM; Dumke et al., 2016, 2020; Fernandez-Huerta et al., 2020b; Chua et al., 2021; Dumke and Spornraft-Ragaller, 2021; Guiraud et al., 2021). Strains with S83N change of ParC are not included. ST, *mgpB* type; MRM, macrolide resistance-associated mutation; and QRM, quinolone resistance-associated mutation.

As regard *M. genitalium*, data about an association between genotypes and clinical aspects of infection are not currently available. After establishing appropriate and comparable methods, more than 20 typing studies have been published and have found many interesting aspects of the epidemiology of the pathogen (Table 2). This aggregation of data includes not only aspects of the transmission of infections among patients with different sexual preferences but also investigated treatment approaches in individual patients that are of great importance for comprehensive evaluation of the clinical management of infections. Overall, these reports confirmed the remarkable genetic diversity of the typing region of the *mgpB* gene among patients of different populations. However, great differences in type frequency have been found. Especially in the risk group of MSM, a striking dominance of type 4 strains in different geographic regions was noted (Figure 3A). It remains unclear whether this is a result of the widespread occurrence of this type in a relatively self-contained sexual network (Fernandez-Huerta et al., 2020b) and/or of a selection advantage in the rectal microenvironment (Guiraud et al., 2021). Despite the high discriminatory power of *mgpB* typing, future spread of distinct types in particular populations might require an increased use of the MG_309 method for successful differentiation (e.g., for comparison of first samples and a positive test of cure; Pineiro et al., 2019; Dumke et al., 2020; Dumke and Spornraft-Ragaller, 2021). In addition, type 4 strains have been found to be macrolide resistant in many cases. However, high rates of macrolide resistance are also observed in other genotypes that occur frequently among the different MSM populations but appear to be lower in rarer types (Figure 3B). In contrast, a clear distribution pattern of quinolone resistance among these genotypes is lacking. Independent mutation events can be assumed, suggesting that the development of resistance is multiclonal, which might explain the lack of a correlation between genotypes and resistance. Besides transmission of resistant strains, acquired resistance after drug exposure is important for the spread of unsusceptible types (Pineiro et al., 2019). As discussed for *M. pneumoniae*, acquisition of resistance can be expected for any genotype of *M. genitalium*, and regional differences in prescriptions and consumption of macrolides and quinolones might play an important role in the resistance rate among circulating strains (Kenyon et al., 2021). This is especially the case in risk groups for sexually transmitted infections, who often receive antibiotic therapy against infections with pathogens other than *M. genitalium*. Currently, little is known about the consequences for the fitness of mycoplasma strains of acquisition of antibiotic resistance and this might be an object for future studies (Guiraud et al., 2021). In practice, clinicians should be aware that types with high rates of resistance commonly circulate in risk populations. Analogously to the P1 adhesin of *M. pneumoniae*, MgpB of *M. genitalium* contains repetitive elements and is an immunodominant protein. Different studies have confirmed changes of the gene sequence in the course of infection, which are related to distinct regions of the gene (Iverson-Cabral et al., 2006; Ma et al., 2010; Burgos et al., 2018; Wood et al., 2020). These recombination processes are of importance for the interaction with the host immune system but do not involve the typing region. This part of the protein was found to be antigenic as well as surface-localized but corresponding antibodies were not associated with the inhibition of hemadsorption (Iverson-Cabral et al., 2015; Aparicio et al., 2020). Nevertheless, sequence differences between *mgpB* types result in amino acid changes (Musatovova and Baseman, 2009; Ma et al., 2010; Dumke et al., 2020; Fernandez-Huerta et al., 2020b). Further studies should analyze if these differences or potential conformation changes of MgpB after recombination events in strains with constant *mgpB* type will have an influence on pathogenesis (Wood et al., 2020). Examples of long-term colonization of patients with the same type (Hjorth et al., 2006; Dumke et al., 2020; Dumke and Spornraft-Ragaller, 2021) and the non-reactivity of the conserved N-terminus with antibodies from infected animals (Iverson-Cabral et al., 2015) may suggest that the typing region of MgpB is of limited importance for interaction with the host immune system.

**Table 2:** Results of *Mycoplasma genitalium* typing studies.

Main aspect	Typing method	Patients/country	No. of patients or samples	Main result(s) of the study	Reference
Methology/epidemiology	*mgpB*	Not specified + couples/worldwide	267	First description of *mgpB* typing; usefulness for investigation of sexual networks and treatment failures	Hjorth et al., 2006
*MgpB* recombination	*mgpB*	Women/Kenya	9	Intrastrain *mgpB* heterogeneity due to recombination	Iverson-Cabral et al., 2006
Methology/epidemiology	*mgpB* + VNTR MG_309	Not specified + couples/worldwide	105	Description of MG_309 typing; usefulness of *mgpB* + VNTR MG_309 typing for general epidemiological studies	Ma et al., 2008
Methology/epidemiology	*mgpB* + VNTR MG_309	Men + women with and without symptoms/France + Tunisia	76	Comparison of methods; *mgpB* typing for general epidemiological studies; *mgpB* + VNTR MG_309 typing for sexual-network studies; and MLVA not suitable	Cazanave et al., 2012
Epidemiology	*mgpB*	Women with previous STD and partners/United States	80	Evaluation of sequence variability between strains from partners and occurrence of reinfections	Musatovova and Baseman, 2009
Resistance	*mgpB*	Not specified/France	136	Evaluation of sequence variability, selection for mutation during treatment; and polyclonality of macrolide resistance	Chrisment et al., 2012
Epidemiology	*mgpB*	Women in sexual health and family planning clinics/Guinea-Bissau	30	Diversity of circulating strains	Olsen et al., 2012
Methology	*mgpB* + VNTR MG_309	Not specified/Cuba	12	Importance of typing for documentation of absence of cross-contamination	Mondeja et al., 2013
Resistance/epidemiology	*mgpB* + VNTR MG_309	Men with and without urethritis/United Kingdom	22	Two major clusters of genotypes with macrolide resistance in both clusters	Pond et al., 2014
Resistance/epidemiology	*mgpB* + VNTR MG_309	Men with NGU/Japan	20	Evaluation of genotype variability	Kikuchi et al., 2014
Resistance/epidemiology	*mgpB* + VNTR MG_309	MSM/Germany	19	Evaluation of genotype variability; comparison of first and follow-up samples	Dumke et al., 2016
Resistance/follow-up	*mgpB* + VNTR MG_309	Men (mainly MSM)/Germany	163	Evaluation of genotype variability; comparison of first and follow-up samples	Dumke et al., 2020
Resistance/epidemiology	*mgpB* + VNTR MG_309	Women in antenatal clinics/Solomon Islands	41	Two major clusters of genotypes, strain replacement after mass drug administration for trachoma elimination	Harrison et al., 2019
Resistance/follow-up	*mgpB* + VNTR MG_309	Patients with suspected STD/Spain	79	Differentiation of persistent and recurrent infections	Pineiro et al., 2019
Resistance/epidemiology	*mgpB* + VNTR MG_309	Heterosexual couples/US	33	Concordance of strains in couples	Xiao et al., 2019
Resistance/epidemiology	*mgpB* + VNTR MG_309	Mainly MSM/Spain	54	Two major clusters of genotypes with correlation to sexual networks and to macrolide resistance	Fernandez-Huerta et al., 2020b
Methology/epidemiology	*mgpB*	Patients of a sexual health center/Australia	52	Establishment of a custom amplicon sequencing approach for *mgpB* typing	Plummer et al., 2020
Resistance/epidemiology	*mgpB*	Not specified/Australia	89	Genotype variability correlated with *de novo* acquisition of resistance	Sweeney et al., 2020
Resistance/epidemiology	*mgpB*	Men in STD clinics/France	78	Lower diversity of types among macrolide-resistant strains	Guiraud et al., 2021
Epidemiology	*mgpB* + VNTR MG_309	Men and women with and without symptoms/South Africa	38	Circulation of different genotypes without geographic clustering	Laumen et al., 2021
Resistance/follow-up	*mgpB* + VNTR MG_309	Mainly MSM/Germany	54	Evaluation of first and follow-up samples during a resistance-guided treatment regime; two major clusters of genotypes with correlation to MSM and macrolide resistance	Dumke and Spornraft-Ragaller, 2021
Resistance/epidemiology	*mgpB*	Asymptomatic MSM/Australia	94	Resistance not restricted to specific genotypes	Chua et al., 2021

STD, sexually transmitted disease; VNTR MG_309, variable number of tandem repeat in gene MG_309; NGU, non-gonococcal urethritis; and MSM, men who have sex with men.

## Conclusion

**Figure 4:**
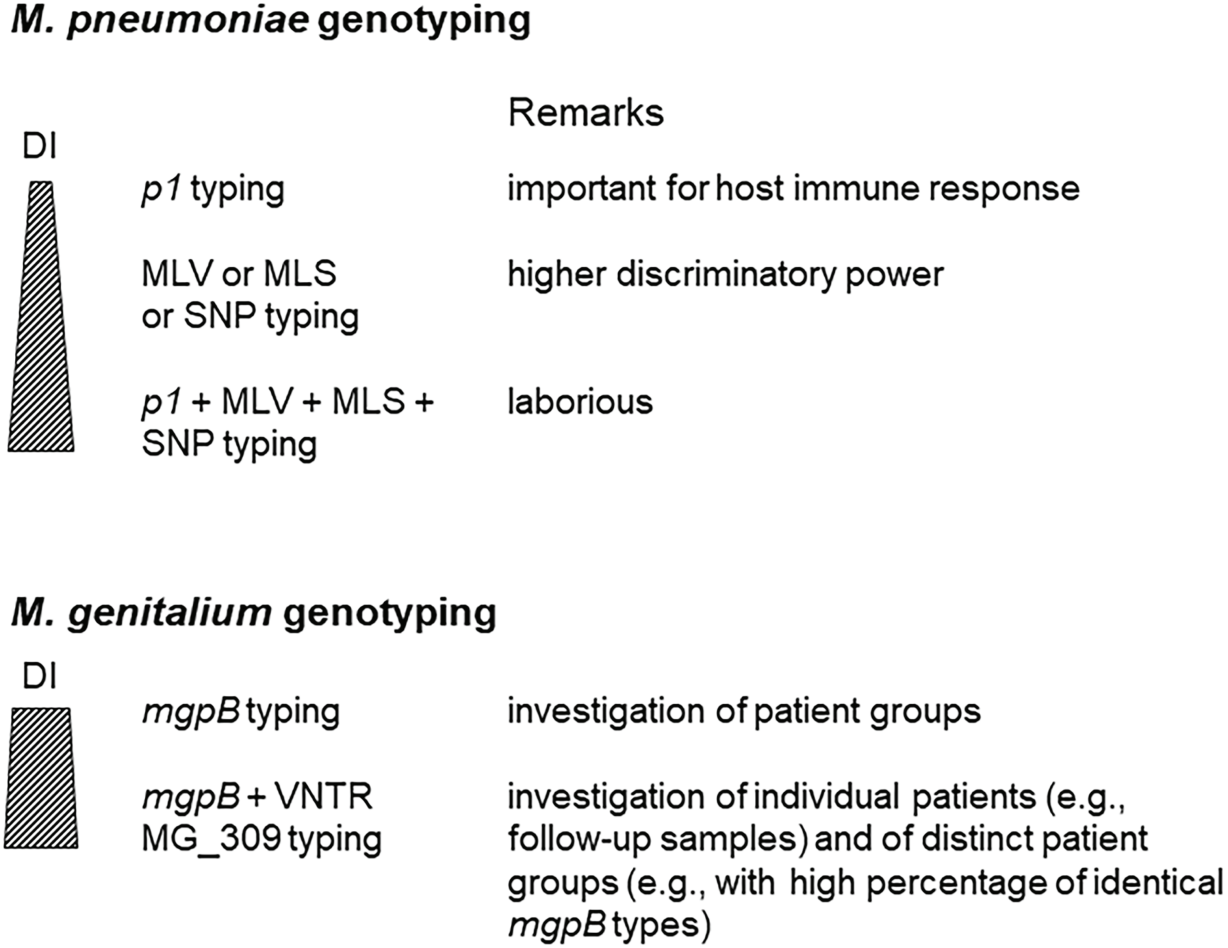
Use of different approaches for *Mycoplasma pneumoniae* and *Mycoplasma genitalium* typing. DI, discriminatory index.

Although whole-genome sequencing (WGS) is likely to replace current methods for molecular typing of *M. pneumoniae* and *M. genitalium*, the simplicity of these assays suggests that they may still have considerable value for epidemiological investigations. This is especially the case if isolates are not available as expected outside of reference laboratories. Whereas the discriminatory power of *mgpB*/MG_309 characterization of *M. genitalium* is high enough for successful differentiation of strains in sexual networks as well as in individual patients, several methods must be combined to reach a DI ≥ 0.9 for typing of *M. pneumoniae* strains (Figure 4). In contrast to the variability of the typing region in the MgpB adhesin of *M. genitalium*, the occurrence of *p1* types in *M. pneumoniae* seems naturally limited by the formation of a functional adhesion complex. For this pathogen, optimization of alternative typing approaches, such as an increase of loci for MLVA, has the potential to enhance their actual discriminatory power. Further studies are needed to evaluate the potential of combinations of current approaches to set internationally accepted recommendations for DI requirement of *M. pneumoniae* typing. At present, only limited knowledge is available regarding the correlation of genotypes with clinical aspects of infections caused by *M. pneumoniae* and *M. genitalium*. These include an association with high regional or population-specific rates of resistance.

## Author Contributions

The author confirms being the sole contributor of this work and has approved it for publication.

## Conflict of Interest

The author declares that the research was conducted in the absence of any commercial or financial relationships that could be construed as a potential conflict of interest.

## Publisher’s Note

All claims expressed in this article are solely those of the authors and do not necessarily represent those of their affiliated organizations, or those of the publisher, the editors and the reviewers. Any product that may be evaluated in this article, or claim that may be made by its manufacturer, is not guaranteed or endorsed by the publisher.

## Footnotes

^1^ http://pubmlst.org/mpneumoniae
